# Does Intranasal Esketamine Show Differential Efficacy with SSRIs versus SNRIs in Treatment-Resistant Depression?

**DOI:** 10.1192/j.eurpsy.2026.11432

**Published:** 2026-07-31

**Authors:** M. Pompili, M. A. Trocchia, G. Di Biase, P. de Roberto, E. Dispenza, S. Sarubbi, D. Erbuto, I. Berardelli

**Affiliations:** 1 Department of Neurosciences, Mental Health and Sensory Organs, Faculty of Medicine and Psychology, Suicide Prevention Centre, Sant ‘Andrea Hospital, Sapienza University of Rome; 2 Psychiatry Residency Training Program, Faculty of Medicine and Psychology, Sapienza University of Rome, Psychiatry Unit, Sant’Andrea Hospital, Rome, Italy

## Abstract

**Introduction:**

Treatment-resistant depression (TRD) represents a major challenge in psychiatry. Nearly one-third of patients with major depressive disorder fail to achieve remission despite adequate antidepressant therapy, and about 30% attempt suicide during their lifetime. Given this elevated risk, identifying treatments that rapidly reduce depressive symptoms and suicidality while improving functioning is crucial. Esketamine, the S-enantiomer of ketamine, is a non-competitive NMDA receptor antagonist enhancing glutamatergic transmission, synaptogenesis, and brain-derived neurotrophic factor (BDNF) release. Its rapid antidepressant and anti-suicidal effects have been demonstrated in randomized controlled trials; however, real-world evidence remains limited, particularly on longitudinal effects on hopelessness, suicidality, and functioning.

**Objectives:**

The study evaluated, in a real-world outpatient setting, the longitudinal efficacy and tolerability of intranasal esketamine in patients with TRD and its impact on hopelessness, suicidal ideation, and functional recovery. A secondary objective was to compare outcomes by concomitant antidepressant class (SSRI vs SNRI) and to provide insight into the clinical and psychopathological mechanisms underlying improvement over time.

**Methods:**

Twenty-seven outpatients with TRD received intranasal esketamine plus an SSRI or SNRI according to clinical indication. Depressive symptoms (Montgomery–Åsberg Depression Rating Scale, MADRS; Beck Depression Inventory-II, BDI-II), suicide risk (Columbia-Suicide Severity Rating Scale, C-SSRS), and hopelessness (Beck Hopelessness Scale, BHS) were assessed. A mixed-model for repeated measures (MMRM) evaluated changes from baseline (T0) to 3-month (T1) and 6-month (T2) follow-ups. For dichotomous variables such as suicidal ideation and adverse effects, Cochran’s Q tests with Bonferroni-corrected pairwise comparisons were applied.

**Results:**

Depressive symptoms decreased significantly over time (MADRS: 33.38→21.43→13.88, p<.001). BDI-II scores declined from 42.62 to 29.19 and 23.34 (p<.001). Hopelessness also decreased (BHS: 16.62→12.31, p<.001). The proportion of patients with suicidal ideation fell from 55.6% to 14% (Q^2^=11.14, p=.004), while suicidal intensity showed a non-significant downward trend (p=.06). No significant interaction between time and antidepressant class emerged. Esketamine was well tolerated; transient dissociative and cardiovascular effects resolved within 40 minutes, and one patient discontinued due to side effects. Seven participants (25.9%) discontinued between 3 and 6 months, mainly for clinical or logistic reasons, with no baseline differences from completers.

**Conclusions:**

After six months, intranasal esketamine was associated with reductions in depressive symptoms, hopelessness, and suicidal ideation, along with good tolerability. No differences in outcomes were found between SSRI and SNRI subgroups.

**Disclosure of Interest:**

None Declared

